# Early Childhood Education in Brazil: Child Rights to ECE in Context of Great Disparities

**DOI:** 10.3390/children10060919

**Published:** 2023-05-24

**Authors:** Abbie Raikes, Jem Heinzel-Nelson Alvarenga Lima, Beatriz Abuchaim

**Affiliations:** 1 College of Public Health, Omaha Campus, University of Nebraska Medical Center, Omaha, NE 68198, USA; 2 ECD Measure, Omaha, NE 68132, USA; 3 Fundação Maria Cecilia Souto Vidigal, São Paulo 04536-010, Brazil

**Keywords:** Brazil, early childhood, education, disparities, preschool, quality

## Abstract

One of the world’s largest countries, Brazil’s national policies on early childhood are some of the most progressive and comprehensive in the world. Notable themes in Brazil’s early childhood system include the national protection of children’s rights, the integration of children’s development, starting at birth, into the national education system, and universal preschool education. These rights are juxtaposed against a highly devolved political structure in the context of significant socioeconomic, racial/ethnic, and geographic disparities. As a result, there is variability in access to quality early childhood settings. This case study explores access to quality early childhood education (ECE) for children aged four to six years. We describe the economic and policy contexts of ECE in Brazil, with emphasis on the role of ECE in addressing regional, racial/ethnic, and economic disparities.

## 1. Early Childhood Education in Brazil: Child Rights to ECE in a Context of Great Disparities

Occupying half of South America’s land mass, Brazil is the giant of the South American continent in size, population, and economy. It is the fifth largest country in the world by area and the sixth most populous with 211 million people [1]. It is considered an upper-middle income country with a gross domestic product (GDP) of $1.86 trillion, making it the ninth largest economy in the world and the largest economy in South America [1]. From 2000 to 2012, Brazil was one of the fastest growing major economies in the world, with an average GDP growth rate of over 5% [1]. Since 2000, Brazil has made tremendous progress in improving the quality of life for children, with substantial declines in infant mortality and child poverty [1]. By 2019, 99% of children survived until the age of five, and 91% of children under five were not stunted, better than average for Latin America and the Caribbean [2]. Ninety-nine percent of the relevant age cohort entered the first grade in 2018 [1].

Brazilian early childhood policies are considered some of the most advanced in the world [3]. Brazil’s vision for early childhood education, care and development is grounded in children’s rights, beginning at birth. Brazil’s Federal Constitution of 1988 articulates the high priority of children’s experiences and affirms early childhood education as a right with an obligation by the government to provide it [4]. This guarantee of children’s rights is one of the strongest governmental protections of rights in the world. Brazil ratified the United Nations Convention on the Rights of the Child in 1996, and, since then, has further protected children’s rights through the 2016 Legal Framework for Early Childhood. The 2016 Legal Framework for Early Childhood guarantees children’s rights beginning in gestation, including rights to health, nutrition, education, support for families, play and leisure, healthy communities, and physical environments [5].

The gains in child wellbeing and protection of children’s rights are contrasted against growing concern over the country’s economic future, including the end of rapid growth rates, corruption charges resulting in the conviction of a former president in 2016, sanctions against some of its leading companies, and the COVID-19 pandemic. The internal problems of corruption and political uncertainty have dampened the investment and business environment and Brazil’s economic growth has slowed down dramatically. Even prior to the pandemic, Brazil faced decades of high levels of unemployment and slow growth in average income, especially when compared to other emerging market economies [6]. These trends have notable implications for young children.

The purpose of this case study was to explore access to quality early childhood education (ECE) for four- to six-year-old preschool children in Brazil, emphasizing the juxtaposition between the expansive and progressive legal framework supporting children’s rights and the wide disparities that characterize early childhood in Brazil. Millions of Brazilian young children lack key aspects of nurturing environments (such as attendance at early learning programs, stimulating and supportive parent interactions, early breastfeeding, and no harsh punishment) [7]. At the same time, Brazil’s emphasis on universal access to ECE makes it a global leader.

Drawing on existing work outlining the importance of country systems in supporting ECE (e.g., [8,9]), this case study examines the development and implementation of Brazil’s ECE system. The impacts of COVID-19 on ECE are highlighted, to elucidate the functioning of the ECE system in the wake of a global emergency. We do not extensively address early childhood care and education for infants and toddlers, although we acknowledge that Brazil’s education system conceptualizes learning as beginning in infancy.

This case study utilizes a constructivist approach, with the overall purpose of integrating multiple data sources to provide a holistic perspective on how guarantees to universal early childhood care and education are implemented in one country, Brazil. Drawing on case study theory [10], a case study approach was selected because the complex social conditions that surround the universal guarantee to early childhood education necessitate a strong focus on context. Our unit of analysis for the case study was the preschool education system in Brazil, and our goal for the case study was to integrate multiple sources of evidence, both quantitative and qualitative, to describe the realities of implementing a rights-based approach in a highly decentralized, unequal country (e.g., [11]). Our research question, which summarizes these priorities, was articulated as follows: How has the universal guarantee to early childhood preschool education been implemented in Brazil in the last decade, and how successful has it been in addressing inequities in access to quality preschool education throughout Brazil? This case study takes the form of a documentary analysis [12], focused on describing the preschool system, emphasizing the use of multiple sources to examine access to quality early childhood education. 

We first provide an overview of the enormity of, and complexities of, the Brazilian education system as a whole and, then, focus on issues related to its access, financing, quality and monitoring. Teacher training, in light of the impacts of COVID-19 on young children and ECE in Brazil, is also addressed.

### 1.1. Socio-Economic Disparities in Brazil

The disparity between rich and poor in Brazil, as measured by the Gini coefficient, is one of the highest in the world [1]. Disparities are evident based on region (Brazil is geopolitically divided into five regions by the Instituto Brasileiro de Geografia e Estatistica (IBGE): north, northeast, southeast, south and central–west), race/ethnicity, parental education, and income. States in the south and southeast of Brazil have the highest GDP per capita and, overall, higher levels of human capital, while states in the north and northeast are home to cities with the lowest GDP per capita and the lowest levels of human capital [13]. Race has a more prominent role in explaining income inequality in Brazil than in many other countries in Latin America [14].

The lives of young Brazilian children are deeply affected by geography, racial/ethnic group status, and family income, including disparities in access to healthcare, good nutrition, early education, and supportive, stimulating home environments [15]. Racially based inequities manifest early in life, for example, Afro-descendant women are six percent more likely than white women to be teenaged mothers and are less likely to seek prenatal care [16]. Children growing up in the southern part of Brazil are more likely to have access to nurturing care than children in the northern states, and lack of access to early childhood education is particularly acute in the northern and midwestern regions [15,17].

Disparities have also been documented in the developmental status of young children. Children born in municipalities in the north and northeast of Brazil develop approximately half of their full potential (measured by the World Bank Human Capital Index, which estimates the expected productivity of a child born today by the age of 18 in a context where education and health conditions remain the same), relative to an average child in a municipality in the southeast [13]. Indigenous communities have a much higher prevalence of anemia and stunting compared to the rest of Brazil [18]. Using a representative sample of preschool children in São Paulo, a recent study found that as many as 30% may face social/emotional delays [19]. As many as 80% of Brazilian adolescents report at least one adverse childhood experiences (ACEs) (defined as physical abuse, sexual abuse, physical neglect, emotional neglect, domestic violence, parental separation and parental death), with significantly higher risks of ACEs among non-white children or children from families with low family income and/or low parental education [20]. A population-based study of child development in Ceará found that nearly 60% of preschool children had at least one adverse childhood experience and 9% had developmental delays. Strong positive associations were found between the presence of the adverse childhood experiences and the developmental delays [21].

These disparities are felt acutely within the educational sector, with vast inequalities in educational progress, resources, access, and quality. Children from poorer families, those living in rural areas, and non-white children are most affected by lack of access to education [2]. For example, while primary school dropout rates are close to zero in developed states, like Santa Catarina, Mato Grosso, and Pernambuco, the overall graduation rate for elementary education was only 76% in the state of Sergipe and 77% in the state of Bahia (both states in the northeastern region) in 2014/2015 [22]. Non-white students also face the following significant disadvantages: (i) illiteracy rates are three times greater, (ii) the rate of graduation from basic education is 15% lower [23]), and (iii) there is considerable evidence of racial discrimination in grading [24]. Indigenous populations, in particular, face difficulties accessing schools, with children often having to travel to neighboring communities to access education [18].

### 1.2. Exacerbation of Inequities during COVID

The COVID-19 pandemic has had devastating impacts on children and their families in Brazil, deeply exacerbating pre-existing inequalities. Before the pandemic, an estimated 19% of families were living in poverty [25]. Due to the COVID-19 pandemic triggering a severe recession in Brazil [26], the number of families living in poverty is now estimated at 25.4%, with 7.9% living in extreme poverty [27]. Before the COVID-19 pandemic, Brazil’s progress in child survival had been the fastest, but, since the pandemic, the rate of improvement in child survival in the northeast of Brazil is now stagnant. A 2021 study led by UNICEF showed that 72% of the most vulnerable families with children under five years of age were not able to feed their children due to lack of money for food during the pandemic [3]. The COVID-19 pandemic led to an increase in maternal mortality and a decrease in children’s vaccine coverage [28].

Brazil was among the countries with the longest period of school closure during the COVID-19 pandemic. Schools were closed for an average of 78 weeks (more than two times the international average), one of the longest closures in the world [13]. Between 2019 and 2021, the total proportion of children, aged six and seven, unable to read and write increased by 15% [29] and school dropout was expected to increase by 365% [30]. Learning loss disproportionately affected the poor, as less than 50% of students in less developed regions benefitted from remote learning, versus 92% in richer parts of the country [13]. Consequently, between 2019 and 2021, the percentage of children not able to read and write increased by 17% for the poorest families, but only by 5% for the richest families [29].

The 2021 School Census showed a 7.3 percent decrease in ECE enrollment compared to 2019 [31]. In Rio de Janeiro in 2020, preschool children gained only 65% of the education they would have had in face-to-face interactions, with the most vulnerable children learning only 48% of what they would have learnt under normal conditions [32]. In Sobral, preschoolers learned only 39% of the language skills and 48% of the mathematical skills of preschoolers in 2019, with greater losses, in terms of school dropouts and learning deficits, for the most vulnerable children [33].

The impacts of COVID-19 will be felt within the education system for years to come. The official figures produced by the Brazilian Institute of Geography and Statistics (IBGE) and the National School Census have not yet captured the expected consequences of school closures and, thus, it is not yet possible to measure the effects caused by the adoption of remote learning (albeit particularly precarious for young children), especially for the most vulnerable portion of the population who did not have access to virtual learning.

## 2. Country Vision for Young Children: Protection of Child Rights and Access to Education Early Childhood Policies

Brazil has a long and distinguished history of innovative approaches to pedagogy. For example, the philosopher and educator Paulo Freire was influenced by the enormous disparities that arose in Brazil due to colonization and autocratic rule, and developed theories of pedagogy designed to support effective engagement in civic life and democracy [34], which, in turn, have influenced emphasis on the development of children’s autonomy and engagement with their environments in the goals and vision for early childhood education. The origins of Brazil’s early childhood system were also influenced by the need to address rampant poverty in the 19th and early 20th centuries [35]. Over the last 40 years, Brazil has made significant policy advances in early childhood education. These advances are clearly linked to expansions in access to early childhood programs, with a shift from viewing early childhood programs as a compensatory mechanism for the most vulnerable to universal access for all children. 

In 1975, the Pre-School Education Coordination Body was established at the federal level, putting the education of four- to six-year-olds under the responsibility of the Ministry of Education. During the 1970s and 1980s, public investments increased in preschools in order to prepare children for primary school and to decrease the repetition and dropout rates at the primary level. In parallel, feminist movements grew in Brazil, promoting women’s rights to join the workforce and to have a place for their children to stay during working hours. The Federal Constitution of 1988 declared early childhood education to be a “right”, its provision a “duty of the State”, and formally identified the Ministry of Education as responsible for the education of all children between the ages of zero to six [36]. In 1996, the Law of Directives and Bases of National Education (Lei de Diretrizes e Bases da Educação Nacional-LDB) defined early childhood education as being a part of basic education, made age seven the starting age for compulsory education, and designated early childhood education as a responsibility of municipal governments [37]. A 2005 amendment to the LDB lowered the compulsory education starting age to six years and, in 2009, a constitutional amendment reduced it to four years (schools were given until 2016 to adapt to the new requirements), making Brazil’s compulsory starting age among the youngest in the world [38]. The 2014–2021 National Education Plan stated that, by 2016, all children of four and five years of age would have access to two years of preschool, officializing the final policy step towards mandating universal preschool [39]. A summary of the policies leading to universal preschool coverage can be seen in Table 1. Consistent with this vision, Brazil provides free care and education for children starting in infancy and extending throughout childhood and adolescence. Education is mandatory from age 4 to 17 years.

### Brazilian Education System

The education system in Brazil is divided into two main levels of education: Basic Education and Higher Education. Compulsory basic education, provided free of charge at public schools, has, in recent years, been extended to early childhood and secondary education (the law was changed in 2009 and schools were given until 2016 to adapt to the new requirements). This change has led to a dramatic increase in access to early childhood education over the past decade with uneven attention to quality [32]. The basic education cycle comprises 14 years broken down into three stages: early childhood education/preschools (ages 4–5); primary or fundamental education (grades 1–9); and secondary education/high school (grades 10–12). There are approximately 48.5 million children enrolled in basic education, 81% of whom attend public schools [40]. Higher education encompasses any education beyond grade 12.

At 6.2% of GDP, Brazil spends much more on education as a whole than the average OECD country and much more than many middle-income countries [1]. However, learning outcomes remain poor. Despite significant increases in access, since the mid-1990s, and improvements in quality, during the first decade of the 2000s, Brazilian students, on average, still perform below most participating countries in internationally comparable tests of mathematics and reading proficiencies. Results from the 2018 PISA (OECD’s Programme for International Student Assessment) show that Brazil is among the countries with the lowest levels of performance. In mathematics, Brazil ranked 70th out of the 77 countries who participated and 57th in reading [41]. According to Brazil’s own assessment, the Basic Education Evaluation System (SAEB), in 2015 only 43% of students met learning standards in mathematics and 55% in Portuguese by the end of fifth grade [40]. These percentages only get worse at the higher stages: 18% in mathematics and 34% in Portuguese in ninth grade and 7% in mathematics and 28% in Portuguese in the last year of high school. Many students have already given up on formal education altogether by the time the last year of schooling is reached [40]. In 2017, only 59% of 19-year-olds had graduated high school [42].

Education is the shared responsibility of the federal, state, and municipal governments (Table 2). Whereas the national government sets overall education policies and is responsible for higher education, basic education is administered locally by the states and municipalities. The core school curriculum, for example, is set nationally, but states and municipalities have the right to adapt it to suit local needs. The main federal supervisory authority in the school system is the National Education Council (Conselho Nacional de Educação, Brasilia, Brazil), an agency of the Ministry of Education. ECE services are under the direct control of municipalities, numbering approximately 5570 [4].

Although Brazil has progressive national policies in place to support access to quality ECE, the quantity and diversity of municipalities throughout Brazil leads to variability across regions in ECE access and quality, which is explored in more depth in the section below. This diversity contributes to geographic and other variations in parents’ approaches to childrearing, such as the use of sibling care as a primary form of childcare and parents’ messages regarding autonomy and relatedness (for example, the extent to which parents value children’s independence versus emphasis on obedience) [43]. These approaches are also associated with economic disparities within Brazil. The regions with frequent reliance on sibling care, for example, are also the regions with more rural populations and more inconsistent access to quality ECE. These disparities in access to ECE compound early inequities, as the most at-risk children have the least access to services. As access increases, assessing the quality of early childhood care and education becomes critical. Given the strong legal framework that guarantees children’s access to ECE, Brazil now faces the task of implementing the legal framework through scaling quality programs [44].

## 3. Universal Access to ECE: Successes and Lingering Disparities

**Figure 1:**
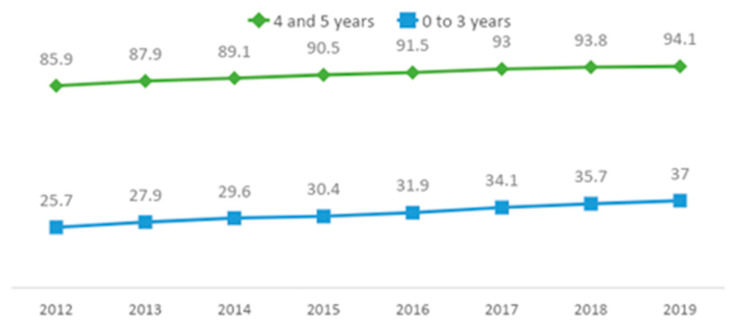
Percentage of Children Enrolled in Preschools and Creches (childcare) 2012–2019 [17].

Prior to the Federal Constitution of 1988, participation in early childhood education in Brazil was low, especially among low-income households. Several states, particularly those in the north and northeast regions, were financially ill-prepared to provide ECE when universal access policies were put into place in the 1990s. However, the introduction of mandatory pre-school education and investments in infrastructure and human resources have led to a tremendous increase in ECE enrollment over recent years with almost 95% of children of ages 4–6 years enrolled in preschool (Figure 1), which is on a par with high-income countries. In the past ten years, gaps in ECE access, related to gender, race, region, and socioeconomic status, have closed significantly.

**Figure 2:**
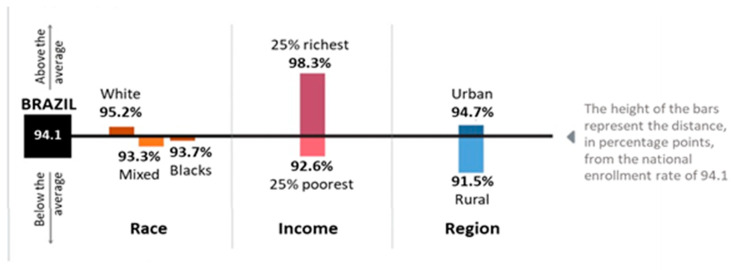
Percentage of Children Enrolled in Preschool by Race, Income, and Region [17].

There is no evidence of unequal access to preschool for boys versus girls [17]. White, black, and mixed children have similar levels of access to preschool and children from both urban and rural households have overall similar levels of access. In Brazil, the IBGE uses five racial classifications in Brazilian censuses: pardo, loosely meaning brown or mixed race, preto (black), branco (white), amarelo (Asian) and indio (Indian/Native). The term pardo can have several meanings including brown, mulatto, mestizo, or any combination of mixed races. Brazilian Indigenous people make up 0.4% of the Brazilian population. About 1% of all children of ages 4–5 years enrolled in preschool identify as Indigenous [17]. While children from better economic backgrounds have slightly greater opportunities to access preschool than children from the poorest households, this gap has closed since 2015 and nearly 93% of children from the poorest households attend ECE, up from 75% (Figure 2) [45].

The National Education Plan stated that 100% of children aged four to six years should have access to early childhood care and education by 2016. While gains are evident (from 86% in 2012 to 94.1% in 2019—see Figure 1), the goal of 100% access has not yet been reached [17]. For younger children, aged from birth to three years, only 37% have access to childcare, with almost twice as many children from the wealthiest families attending childcare (54%) as those from the poorest households (28%) [17]. When broken down by age group, 16% of children from birth to one year of age are enrolled in childcare while 58% of children two to three years of age are enrolled in childcare. The National Education Plan states that, by 2024, 50% of children from birth to three years should have access to childcare. At present, though, waiting lists for childcare are long in urban areas and coverage is uneven across Brazil.

### 3.1. Disparities in Access to ECE

**Figure 3:**
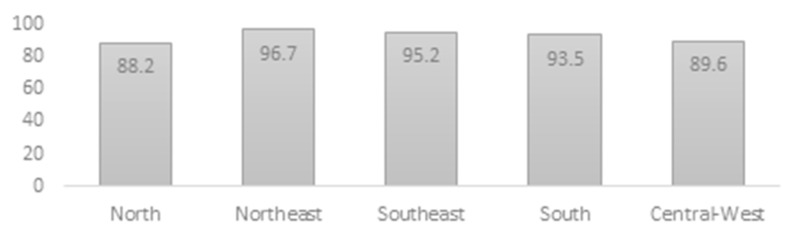
ECE Access for Children of ages 4–5 by region in 2019 [45].

While ECE access overall is high across all regions (Figure 3), rates of enrollment mask differences of access between states within the same region. For example, Amapá, located in the northern region, is the second least populated state in Brazil with most of its population living in poverty (0.4% of the Brazilian population; responsible for only 0.22% of the Brazilian GDP) [17]. Amapá has preschool enrollment rates of 75.1% [17]. In comparison, Tocantins, also a state in the same northern region, has enrollment rates of 94% [17]. Table 3 lists all 26 states within Brazil and their preschool enrollment rates from 2012–2019.

**Figure 4:**
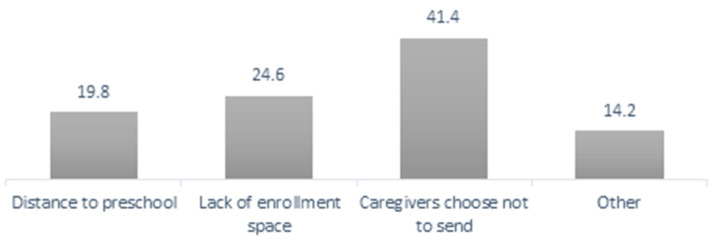
Reasons for Not Attending Preschool, Children of age 4–6 years [17].

In 2019, there were approximately 316,500 children of ages four to five years who did not attend preschool [17]. A national survey administered in 2017 found that most of these children did not attend preschool because the caregiver chose not to send them [17] (Figure 4). In addition, a recent study showed that poor and/or black children, whose mothers were single, aged 19 years or less, had low education and/or informal jobs, faced higher risks of not attending preschool. In the midwestern region only 80% of black children were enrolled in preschool, compared to 89% of white children in the same region, or 93.5% of white children in Brazil [46]. 

Eighty percent of children in ECE in 2019 attended public institutions [17]. Some work suggests that quality is lower in public ECE than in private ECE, contributing to an unequal system that continues to privilege wealthy children [47]. In primary grades, the largest disparities in learning outcomes exist between children who attend private schools in industrialized and developed states and children who attend public schools in impoverished and more rural states [48].

### 3.2. Access for Children with Special Needs

**Figure 5:**
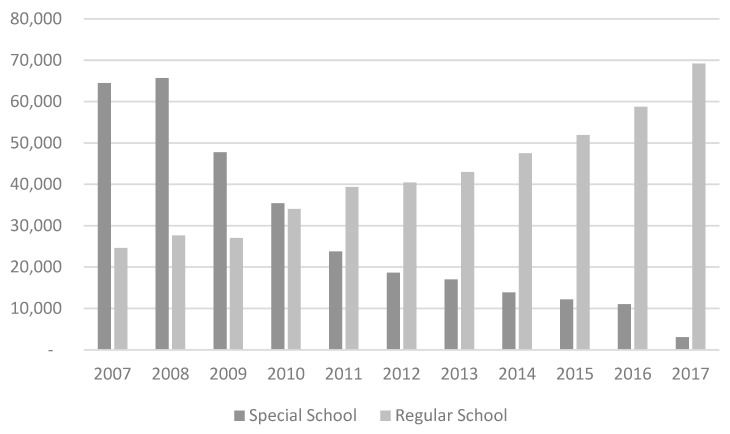
Number of Children Enrolled in ECE Programs by Type of School (Typical School Compared to Specialized Schools) (2007–2017) [50].

The Child and Adolescent Statue (1990) and the National Policy on Special Education (1994) indicate that children with special needs should be attended to in the regular network of childcare centers and preschools (instead of in specialized or separate schools for those with special needs). In 2015, in conjunction with the Lei Brasileira de Inclusão da Pessoa com Deficiência (n. 13.146/2015) [49] the Ministry of Education released a joint technical note on Special Education Guidelines for Early Childhood Education. This law ensures that children with special needs are included in regular public and private early childhood schools, and have access to assistive technology. The law provides guidelines for teachers in inclusion practices. The law states that schools must provide special training for teachers and hire assistants to support students with special needs and that the curricula and pedagogical approaches must be adapted according to the individual needs of students. As seen in Figure 5, over the last decade, due to the inclusion policy in force, there has been a significant evolution in the enrollment of children with special needs directly into common classes/schools (as opposed to specialized schools) for early childhood education [50].

## 4. Financing and Inequalities of ECE

Sustained public financial support is critical for the growth and quality of ECE programs. In 2016, the Brazilian government spent about 0.7% of its GDP on early childhood education, in line with the OECD average [51]. However, given the country’s lower GDP per capita, spending per child is only USD 3700, one of the lowest across OECD countries and less than half the OECD average of USD 7800 [51]. States and municipalities are constitutionally mandated to spend at least one quarter of their tax revenues on education, while the federal government is required to spend at least 18% of its tax income on education [4]. In addition, there are spending targets for specific sectors. For example, a 1996 constitutional amendment required states and municipalities to spend at least 60% of their education budget on elementary education—a requirement that helped make elementary education universal in Brazil [52].

However, municipalities have wide discretion on how much to specifically invest in ECE. As a result, wealthier municipalities tend to invest more in preschools than do poorer municipalities. In addition, the federal funding formula distributes money evenly among wealthy and poor states, even if some states do not need as much federal support [52]. Not surprisingly, major regional differences in the investment in early childhood education are readily apparent. Brazil’s southeast region, for example, spends four times more per pupil on public preschool education than the relatively poor northeast region does [53].

The Fund for the Maintenance and Development of Basic Education (FUNDEB) is the main financing mechanism for public education in Brazil, benefitting over 38 million students enrolled in public schools at all levels, pre-school, primary, and secondary [54]. The government financing mechanism ensures marginalized schools have adequate resources. FUNDEB is designed to broaden access to early childhood, elementary, and secondary education in rural areas, in underserved regions, and among indigenous populations. State funds for basic education are provided, in part, from the income generated by various taxes. FUNDEB helps to offset poorer states that do not have as much income from taxes as compared to wealthier states. The fund guarantees a minimum amount to be invested, per student, in every municipality in the country.

In 2020, a constitutional amendment increased the amount the federal government contributes to FUNDEB from 10% to 23% [54,55]. Funding increased to R17.5 billion (US$350 million) in 2021 and is expected to reach R39.3 billion (US$786 million) by 2026. Under the new amendment, resources are also now transferred directly to the poorest municipalities, instead of to the states, making educational investments more equitable. This new law (Lei 14.113) makes FUNDEB a permanent funding mechanism for education in Brazil, and is an important milestone in guaranteeing access to public services for all children and youths. Currently, congress and government are debating strategies to regulate and implement what is being called the “new FUNDEB.” The discussion of the new FUNDEB raises a crucial point for ECE, namely, that the amount of money spent on ECE is not enough to reach the minimum standards of quality. Investment should be at least three times higher than what the average municipality currently spends per capita [36].

In addition to FUNDEB, the National Government provides additional funding mechanisms to municipalities and states. The Fundo Nacional para o Desenvolvimento da Educação (FNDE) is responsible for programs aimed at supporting local governments. The FNDE provides assistance for the acquisition and improvement of transportation, food, materials, equipment, books, and professional development at the local level. There are ECE-specific programs within FNDE. One example is “Proinfância”, which aims to fund the construction of new buildings for ECE centers and the acquisition of furniture and materials to help municipalities expand access to ECE. This program started in 2007 but has faced significant implementational challenges (for instance, excessive bureaucracy, cases of corruption, difficulty in meeting technical specifications, inadequacy of lands, etc.). As of 2020, more than 15,000 construction projects for early childhood and primary education had been funded and initiated under Proinfância, but only 50% of these had actually been completed [56].

Beyond financing for ECE, Brazil has attempted to address some of the inequities related to young children’s development and learning and home environments through direct cash payments. Documented improvements in the wellbeing of children have been attributed to the government’s support for families, through programs such as Bolsa Familia, a conditional cash transfer program for families with young children [43]. Bolsa Familia has demonstrated positive impacts, including reduced child mortality, increased access to health care and food, and reduced school dropouts [57]. In 2021, the national government ended Bolsa Família and created a similar program called Auxílio Brasil, under which the amounts of cash transfer are larger per capita than they were under Bolsa Familia. The main difference between these two programs is that Auxílio Brazil is limited to poor families with pregnant women or people until 21 years of age, whereas Bolsa Familia had wider coverage. The conditionalities are similar between the two programs, such as school attendance for children at a mandatory age, children’s immunizations, doctor’s appointments for young children and pregnant women, among others. As of August 2022, 5.7 million Brazilian families received benefits, which are thought to play an important role in decreasing poverty and increasing purchasing power and employability, although the results are not conclusive [58] and no rigorous external evaluation has been reported.

## 5. ECE Curriculum

Although the official responsibility for implementing early childhood education policies falls on the municipal government, the Brazilian federal government provides technical and financial support to guarantee that minimum quality standards are achieved in each Brazilian school. Instituted in 1999, and revised in 2009, the National Curricular Guidelines for Early Childhood Education (Diretrizes Curriculares Nacionais para a Educação Infantil—DCNEI,) is mandatory for all public and private ECE centers. The main goal of the DCNEI is to guide public policies and the development, planning, implementation and evaluation of pedagogical and curricular activities for early childhood education. The document outlines the basic parameters and key ECE learning experiences to be included in the development of pedagogical plans by ECE institutions, including teaching strategies, organization of physical space, and a general calendar [59]. The DCNEI also recognizes the value of assessing and documenting the care and education practices for children and recommended that a formal monitoring system be established. Although the DCNEI is considered a legal document, in practice this document is rarely used by ECE institutions as a basis or guide in developing their pedagogical plans and there is no system in place to ensure its use [36].

In 2017, the National Common Core Curriculum (BNCC) was approved by the National Educational Council (CNE). The BNCC sets the core content and modalities of education for the entire country. It defines the learning rights and goals for all three levels within the basic education system: early childhood education, fundamental education, and high school education [60]. The 2017 BNCC was the first time ECE was included in the formal basic education curriculum and identifies the following overarching learning goals for ECE:

Get along with other children and adults, developing a respect for other cultures and differences between people;

Learn through play;

Actively participate and have choice in activities;

Explore and learn concepts of art, language, mathematics, science and technology through multiple means and modalities and in various contexts;

Learn to express oneself, through developing opinions and asking questions; and

Develop a positive image of oneself and others.

The BNCC ECE curriculum structure builds on the learning experiences identified in the DCNEI as crucial for ECE. The BNCC ECE avoids curriculum organization using formal discipline/subject areas. Instead, the key learning experiences taken from the DCNEI are structured under the following five “Fields of Experience”, acknowledging the transdisciplinary nature of children’s learning through play and interactions:

The I, the Other, and the Us

Body, Gestures, and Movements

Listening, Speaking, Thinking, and Imagining

Traces, Sounds, Colors and Images

Space, Time, Relationships, and Transformations

Learning objectives in each of the five Fields of Experiences are defined for three key age groups: infants (from 0 to 18 months); toddlers (from 19 months to 3 years) and preschoolers from 4 to 5 years). While the national core curriculum is developed at the federal level, municipalities have the right to adapt it to suit their local needs. In 2019, municipalities began the process of adapting the BNCC ECE into their programs and teacher training and by 2020 it was expected that the BNCC ECE would be used in all classrooms. By 2022, 99% of Brazilian municipalities had a local curriculum document aligned with the BNCC ECE [61].

The BNCC ECE is considered a key advancement for the quality of ECE in Brazil as it defines the progression of learning outcomes and standards for children of 0–5 years of age. As mentioned earlier, the general curriculum concepts, as stated in the DCNEI, were not consistently used in practice in Brazilian ECE centers. The inclusion of clear learning objectives for early childhood within the BNCC aims to promote higher quality curriculums, materials, and teaching to carry through into the first stage of Basic Education [40]. As the BNCC is scaled, it is crucial to evaluate how well it is being implemented in schools.

## 6. Monitoring ECE Quality

There are no guidelines on assessment or evaluation within the curriculum document and there are no systemic national efforts to assess implementation of quality ECE [62] (Operrti et al., 2018). In 2006 (and updated in 2018), the federal government published the “Quality Standards for Early Childhood Education” [63]. This non-binding document outlines the standards for ECE under the following eight key areas:

Management of Education System;

Teacher Training,

Career and Compensation;

Management of ECE Institutions;

Curriculum, Interactions and Pedagogical approaches;

Interactions with Family and Community;

Intersectoral Coordination;

Space, Materials and Furniture; and

Infrastructure.

Based on the Quality Standards, the Ministry of Education developed “Monitoring the Use of ECE Quality Indicators” [64], a self-assessment for individual ECE centers to measure compliance with the above-mentioned ECE quality standards. Results from the ECE Quality Indicators are intended to guide improvements within ECE facilities, rather than inform higher-level public policy development [65].

One of the key challenges in ensuring the quality of ECE in Brazil is the overall lack of a formal national evaluation system to support the use of the BNCC, measure early childhood education outcomes, and measure ECE quality [65]. Compared to other ECE public policies, such as teacher training, curriculum implementation, and financing, the integration of measurement (for both program quality and child assessment) has experienced the least amount of progress [66]. The Plano Nacional de Educação (2014-24) stated that ECE should have had a national assessment starting in 2015. However, it was only in 2021 that ECE was included in the National Education Assessment System (Sistema Nacional de Avaliação Educacional-Saeb), which is administered in all public schools.

In 2018, the Ministry of Education announced plans for the development of a national ECE assessment system. In 2019, the ECE assessment was piloted as part of the Saeb and in November 2021, ECE was formally included in the Saeb, administered to a representative sample of teachers, principals and municipal public managers through electronic questionnaires. The ECE assessment does not focus on the assessment of student’s learning and outcomes but on the conditions of ECE centers, such as infrastructure and human resources. Like other elements of the Saeb, ECE assessment is implemented every two years, via questionnaires based on the 2018 Quality Standards for Early Childhood Education and the BNCC. The questionnaires collect information on pedagogical resources, educator profiles, management, infrastructure, financing, and other key aspects of the ECE centers. The ECE assessment is now included under the jurisdiction of the Instituto Nacional de Estudos e Pesquisas Educacionais Anísio Teixeira (INEP), which is the institution responsible for large scale assessments in Brazil. Initial results from the complete 2021 SAEB were published in September 2022 [67]. However, very little data and information collected from the ECE assessment was published, indicating that this stage of education is still quite nascent.

**Figure 6:**
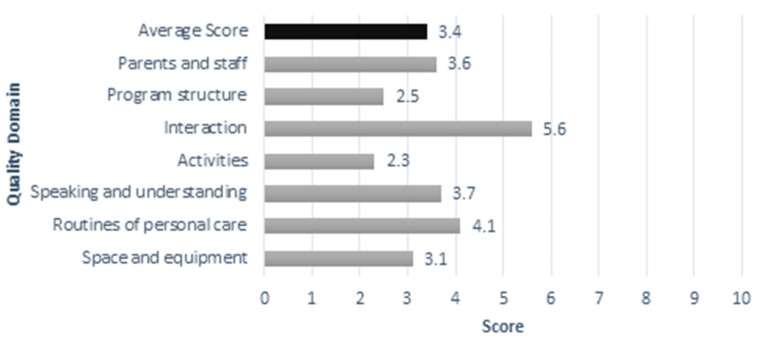
Average ECERS-R Scores in Six State Capitals [68].

While nationally representative data are not available, there have been several small-scale studies implemented in Brazil to measure the quality of ECE. In 2009, the Ministry of Education, with the support of the Carlos Chagas Foundation and the Inter-American Development Bank, measured the quality of 147 preschools in six state capitals (urban cities) using a translated version of the Early Childhood Environment Rating Scale, Revised Edition (ECERS-R) [68](Harms et al., 2005). In general, the results showed very low levels of quality and highlighted differences in infrastructure, daily routines, teacher practices and teacher preparation across regions [38,69]. On a scale from 1–10, the average quality score was 3.4 (see Figure 6). Overall, 30% of all ECEs were rated as inadequate, only 4% achieved a “good” rating, and no institutions achieved an excellent rating [38]. Quality outside the large urban areas is likely to be even lower.

In 2018, an adapted version of the Measuring Early Learning Quality and Outcomes (MELQO) was used in 63 preschools in Boa Vista (a municipality in the north) [70]. Results from the Boa Vista assessment showed significant variation in quality of ECE and children’s learning outcomes within educational systems and municipalities. Urban ECE and ECE in capital cities had higher quality classrooms and better learning outcomes than ECE in rural areas or smaller cities. The average learning difference between children who were enrolled in high-quality ECE and those enrolled in low-quality ECE within the same municipality amounted to the equivalent of 6.37 months in the development of early language and literacy skills and 4.44 months in the development of mathematical skills [71,72].

In 2021, 12 municipalities assessed a sample of ECE using an environmental quality observation instrument—“Escala de Avaliação de Ambientes de Aprendizagens dedicados à Primeira Infância—EAPI”, another adaptation of the MELQO that was aligned with BNCC concepts [28]. The evaluation included 3467 classrooms of children from two to five years of age. The EAPI found that in most observed classrooms, teachers used high quality practices in language, visual arts and conceptions of numbers, space and objects classifications [28]. Only 38% of the classrooms had high-quality reading and writing practices. In 55% of the classrooms, teachers did not read any books to children during the observation period of three and a half hours. Children were allowed to play freely in only 58% of the observed classrooms. Verbal and physical negative interactions between teachers and children were observed in 11% and 2.9% of the classrooms, respectively [28]. These results strongly suggest that the concepts of BNCC are not yet consistently guiding teacher practices in Brazil and teachers require additional training and support to ensure that all children have access to quality ECCE [28].

## 7. Pre-Service and In-Service Training

In 2021, there were 595,000 ECE teachers, 96.3% of whom were women, between 30 and 49 years of age, on average. and with a total of78% holding a university degree related to teaching [31]. Many teachers are non-white and from disadvantaged social and economic backgrounds, 90% of whom studied in low-cost universities [73]. Conditions and pay are worse for ECE teachers than primary and high school teachers. While most ECE teachers receive specific courses related to ECE, the approach to training ECCE teachers is more theoretical than practical, resulting in challenges when recently graduated teachers begin work at schools [74,75,76,77]. Trained teachers generally do not have a consistent knowledge base about child development, curriculum and pedagogical methodologies and are not trained in how to plan, implement, and evaluate pedagogical practice aligned to the BNCC [78,79]. In December 2019, the Ministry of Education and the National Education Council defined new guidelines for teacher pre-service training, aiming to address the main concepts of BNCC, including specifications for ECE. As this is a new regulation, there is not yet any insight on its impacts in pedagogical curricula or teaching within ECE classrooms.

In-service training plays a fundamental role in addressing the BNCC by building competencies in ECE practices [80]. Since early childhood education is the responsibility of municipalities, it is up to the municipalities to develop in-service training programs for teachers in public preschools [36]. However, there are no specific national guidelines about methodologies, program content, or modalities for the in-service training. Municipalities, thus, employ their own strategies to train teachers, leading to notable variations in teacher training. Some municipalities have large events or courses with external professionals, whereas others invest in professionals inside ECEs, such as a pedagogical coordinator, to work closely with teachers in their day-by-day practices. Qualitative studies have shown that the second strategy seems to work better to improve teachers’ performances [36]. At the federal level, there are only a few online in-service courses related to ECE provided by the Ministry of Education, underscoring the overall lack of resources made available to support ECE professionals. Successful implementation of the BNCC ECE curriculum requires reorienting teachers’ beliefs about young children’s development and how they learn. This is especially critical for teacher preparation for working with children from diverse cultural, geographic, and socioeconomic backgrounds [45].

## 8. Engaging Parents and Communities

According to the Lei de Diretrizes e Bases da Educação Nacional, schools are responsible for building solid relationships with communities, informing families about curriculum implementation and student learning outcomes, and engaging communities in decision-making processes [37]. While not mandatory, the law suggests that each school in Brazil should have a School Board to support and monitor schools, composed of the principal, teachers, employees, parents, community leaders, and students, among others. The process to elect the representatives is defined by each school. However, there is no information about the number of school boards in Brazil for any level of education.

In 2004, the Ministry of Education, in partnership with local governments, created a program to support and improve the school boards, publishing materials and offering training for board members. At present, the actions of this program are part of a new program named “Família na escola” (translated as “family at school”). “Família na escola” aims to empower the school boards and publishes materials and strategies to help schools interact more with families. Under the program “Dinheiro direto na escola” (translated as “money directly to school”), the Federal Government transfers money directly to schools and gives them autonomy, along with the school boards, to invest funds, based on local priorities.

The 2016 Legal Framework for Early Childhood emphasizes the importance of programs aimed at strengthening the role of families in the education of their children [5]. However, although outlined in federal law, families’ participation in ECE is not always viewed as relevant. Many ECE programs do not have strong and systematic strategies to engage families, leading to issues in communicating effectively between schools and homes. The poorer the family, the worse these problems become [81]. ECE professionals may have prejudices regarding vulnerable families, believing that more vulnerable parents do not know how to educate a child and do not invest time in them, and poor families may not feel empowered to ask questions or complain about services [81]. Rodrigues and Muanis reviewed a set of 22 papers published in Brazil about ECE schools and families [82]. Most of these publications noted misalignment in views between families and teachers regarding children’s education. Some families reported little interest in learning about what their children did at school, while teachers reported limited availability to talk to families about the development of their children. However, research on parental engagement is limited.

During the COVID-19 crisis, and given prolonged closures, teachers worked more closely with families to coordinate remote learning, and to better understand their home and life conditions and challenges. After facing the difficulties of educating their own children at home, caregivers may have developed a deeper appreciation for schools, which, in turn, may have improved parental engagement going forward [83].

## 9. Challenges and Opportunities

Brazil has made significant gains in its journey towards universal preschool and ensuring access to ECE. This case study documents both the progress forward as well as the challenges in implementing a rights-based approach to early childhood education. The history of Brazil’s pedagogical vision for education in general, and early childhood education in particular, is grounded in the ability of education to transcend structural and political inequities. However, to fully manifest Brazil’s vision for ensuring children’s rights, there is still much to do to ensure access to quality ECE, particularly the most vulnerable. While the 92% enrollment for children of 4–5 years of age is a tremendous accomplishment, the 8% who are excluded are also the most vulnerable. Moving forward, a central priority for manifesting the vision of the Brazilian system will be to increase access and ensure the inclusion of the most vulnerable children within the ECE system. These vulnerable children may have the most to gain [38]. Further, gaps in access for children 0–3 years of age are significant and far from the national target of 50%.

A key challenge for Brazil is to address the inequities that arise due to the autonomy of Brazil’s 5570 municipalities in implementing ECE, which, in turn, leads to fragmented funding. There is a tension point between Brazil’s commitment to universal access to education and the commitment to a highly decentralized approach to governing. Lower levels of investment in the poorer municipalities lead to lower levels of quality and service provision for children. The risk of low quality ECE means that Brazil’s system could fall short of its vision or even perpetuate inequities in lifelong learning and wellbeing. This is a principal challenge to the national ECE policy providing equal access to high-quality services [45].

From this descriptive case study, a focus on ensuring quality ECE emerges as a next step. Brazil has made great gains in improving access to preschools for children across Brazil. A quality assurance framework is now needed to promote regular monitoring and evaluation of preschool quality. While the studies of ECE quality reviewed in this case study were not nationally representative, the findings indicate a strong need for quality improvements to the Brazilian ECE system. Although there is no consolidated system in Brazil that guarantees the quality of services for early childhood education, there are defined standards that are fundamental elements for the creation of a monitoring structure. There are already several measurement tools adapted to Brazil. The challenge now is to design a system that provides periodic measurement, monitoring, and quality improvement, informed by data on quality ECE.

Inclusion of ECE into the formal assessment system is one way to generate concrete data to guide the ECE system. While the inclusion of the ECE assessment into the National Education Assessment System is a step in the right direction, there is still much to be done. Given that the ECE assessment is a self-assessment filled out by teachers, principals, and municipal public managers within their own schools and/or municipalities, there is a high chance for bias in survey results. In addition, this assessment only collects inputs (i.e., number of children, square footage, teacher training received, existence of curriculum, materials, etc.). It does not collect data on child learning or quality of learning experiences. An effective system would consolidate reference documents, monitoring tools and quality standards for preschools to create a framework for municipalities to ensure quality. The federal government should encourage strong municipal monitoring systems that hold preschools accountable for their results and introduce a standard observational tool to allow for regular, systematic monitoring of activity quality and program structure. Nationally representative data on children’s development outcomes would help in monitoring disparities in quality and child outcomes, and, thus, would generate evidence to better identify possible areas for improvements in ECE policies and practices.

Beyond ensuring access to quality ECE, the most vulnerable populations require further protection and support, especially following the COVID-19 pandemic. Brazil has had internationally renowned success with its innovative social policies and programs, such as Bolsa Familia and Auxílio Brasil. These programs have proven to reach those most in need, with positive impacts on early childhood development and education. Moving forward, Brazil can build on its experience in implementing these types of social protection programs to support the most vulnerable children and their families by improving access and quality to early childhood programs.

Overall, Brazil’s progressive policy framework sets the stage for innovation in early childhood. Brazil now has the opportunity to learn from the diversity of ECE policies and programs among states and municipalities. Documenting how and why some municipalities consistently improve early childhood services and replicating their successes would provide unique opportunities to document the importance of quality ECE across Brazil.

Future directions for research include ongoing work to trace the realities of large-scale implementation of Brazil’s innovations in pedagogy and child rights in early childhood programs, so as to more fully understand how the vision for early childhood education can be integrated into the social and political contexts of Brazil. Simply documenting trends in Brazilian early childhood education overlooks the complexity of the history and social contexts [84], underscoring the need for case studies and other approaches to document both the innovations and the challenges of Brazilian ECE.

## Figures and Tables

**Table 1:** Brazil’s Journey to Universal Preschool.

Year	Law	Policy Change
1975	Pre-school Education Coordinating Body	Education of 4- to 6-year-olds put under responsibility of Ministry of Education
1988	Federal Constitution (Art. 208.IV)	Defined ECE (0–6) as government responsibility and a child’s right Set starting age for obligatory education at 7 Placed responsibility for ECE in municipalities
1996	Law of Directives and Bases of National Education	Reaffirmed ECE (0–6) as part of basic education Required that ECE teachers should have a high school or an undergraduate diploma in teaching
2005	Law 11.114	Lowered obligatory education starting age to 6
2006	Law 11.247	Extended Fundamental School to 9 years, with the enrollment of 6-year-old children
2009	Constitutional Amendment 59	Lowered obligatory education starting age to 4 (universal preschool)
2014	2014–2021 National Education Plan	By 2016, schools required to offer 2 years of preschool (enforcing Constitutional Amendment 59)

**Table 2:** Brazil Basic Education System.

Stages	Early Childhood Education		Fundamental School		High School
Modalities	Childcare/Creche	Preschool	Primary School	Middle School	15–17
Age	0–3	4–5	6–10	11–14	
Responsibility	Municipalities		States and Municipalities		States

**Table 3:** Preschool Enrollment Rates across States (2012–2019) [17].

Region/State	2012	2013	2014	2015	2016	2017	2018	2019
**BRAZIL**	**85.9**	**87.9**	**89.1**	**90.5**	**91.5**	**93**	**93.8**	**94.1**
**North Region**	**75**	**78.8**	**80.3**	**80.6**	**86.7**	**86.9**	**88**	**88.2**
Rondônia	67.9	69.7	83.8	81.8	85	87.8	89.6	86.8
Acre	64.4	69.6	73.4	74.2	77.7	81.7	79.9	79.5
Amazonas	71.7	75.9	74.4	75.8	83	81.6	87.8	87.6
Roraima	77.3	82.1	89.9	91.3	93.5	93.1	91.6	89.9
Pará	78.4	82.3	83.7	82.2	89.7	90.7	89.7	90.3
Amapá	63.7	77.1	70	70.2	76.3	72.7	67.8	75.1
Tocantins	83.3	81.3	80.6	91.3	92.7	92.8	93.5	93.9
**Northeast Region**	**90.7**	**92.6**	**92.4**	**94.1**	**94.9**	**95.6**	**96.3**	**96.7**
Maranhão	91.7	92.7	93.8	94.6	97	97.2	97.4	97.6
Piauí	92.7	96.8	96.6	97.1	99.2	97.7	97.1	99.1
Ceará	95	96.8	97.3	95.7	97	98	98.5	97.4
Rio Grande do Norte	93.9	92.5	89.2	96.1	96.6	96.9	97.2	98
Paraíba	89.1	95.1	93.4	91.6	92.1	97	94	95.3
Pernambuco	90.5	88	90.2	94.6	94.6	91.4	93.5	94.5
Alagoas	84.6	83.7	87.5	83.3	88.7	90.2	92.5	93.5
Sergipe	95.3	96.2	91.8	93.3	92.2	94.2	95.2	96.4
Bahia	87.5	92.7	90.7	94.5	93.7	95.9	97.6	97.3
**Southeast Region**	**88.4**	**90.5**	**91.8**	**93**	**91.7**	**94.4**	**94.9**	**95.2**
Minas Gerais	88.1	88.7	90.1	91.7	94	95.1	94.6	96.1
Espírito Santo	93.3	91.2	92.4	91.1	95.5	93.8	96.3	96.1
Rio de Janeiro	88.1	89.8	90.4	93.2	87.1	91.3	92.3	92.6
São Paulo	88.2	91.4	93.1	93.8	92	95.2	95.7	95.6
**South Region**	**80.2**	**80.4**	**85.4**	**86.8**	**90**	**90.4**	**92.5**	**93.5**
Paraná	82.3	85.1	87.5	89.3	92.5	91.3	94.8	94.4
Santa Catarina	89.2	87.8	89.9	94.2	92.8	93.3	94.3	96.5
Rio Grande do Sul	72.3	70.6	80.1	79.6	85.5	87.5	88.9	90.5
**Central-West Region**	**79.7**	**82.5**	**83**	**85**	**86.9**	**88.6**	**89.2**	**89.6**
Mato Grosso do Sul	78.7	84.9	88.8	86.4	89	91.4	91	91.8
Mato Grosso	79.5	80.3	84.1	83.7	85.4	89.1	93	94.1
Goiás	78	81	80	84	89.1	88.1	87.5	86.6
Distrito Federal	84.5	86.6	82.7	87.3	81.7	85.8	86.3	87

## Data Availability

No new data were created or analyzed in this study. Data sharing is not applicable to this article.
